# Multivariable Analysis of Nutritional and Socio-Economic Profiles Shows Differences in Incident Anemia for Northern and Southern Jiangsu in China

**DOI:** 10.3390/nu9101153

**Published:** 2017-10-21

**Authors:** Stefan Mutter, Aaron E. Casey, Shiqi Zhen, Zumin Shi, Ville-Petteri Mäkinen

**Affiliations:** 1 South Australian Health and Medical Research Institute (SAHMRI), Adelaide SA 5000, Australia; stefan.mutter@sahmri.com (S.M.); aaron.casey@sahmri.com (A.E.C.); 2 School of Biological Sciences, University of Adelaide, Adelaide SA 5005, Australia; 3 Department of Nutrition and Foodborne Disease Prevention, Jiangsu Provincial Centre for Disease Control and Prevention, Nanjing 210009, China; cdczsq@163.com; 4 School of Medicine, University of Adelaide, Adelaide SA 5005, Australia; zumin.shi@adelaide.edu.au; 5 Computational Medicine, Faculty of Medicine, University of Oulu and Biocenter Oulu, 90014 Oulu, Finland

**Keywords:** anemia, subtypes, subgroups, China, grains, Jiangsu, geographical divide, iron, rice, wheat

## Abstract

Anemia is a prevalent public health problem associated with nutritional and socio-economic factors that contribute to iron deficiency. To understand the complex interplay of risk factors, we investigated a prospective population sample from the Jiangsu province in China. At baseline, three-day food intake was measured for 2849 individuals (20 to 87 years of age, mean age 47 ± 14, range 20–87 years, 64% women). At a five-year follow-up, anemia status was re-assessed for 1262 individuals. The dataset was split and age-matched to accommodate cross-sectional (*n* = 2526), prospective (*n* = 837), and subgroup designs (*n* = 1844). We applied a machine learning framework (self-organizing map) to define four subgroups. The first two subgroups were primarily from the less affluent North: the High Fibre subgroup had a higher iron intake (35 vs. 21 mg/day) and lower anemia incidence (10% vs. 25%) compared to the Low Vegetable subgroup. However, the predominantly Southern subgroups were surprising: the Low Fibre subgroup showed a lower anemia incidence (10% vs. 27%), yet also a lower iron intake (20 vs. 28 mg/day) compared to the High Rice subgroup. These results suggest that interventions and iron intake guidelines should be tailored to regional, nutritional, and socio-economic subgroups.

## 1. Introduction

Anemia is a condition characterised by the reduction of red blood cell mass which leads to an inferior oxygen supply to tissues [1], affecting 25% of the global population [2] with the highest prevalence in Africa but a larger absolute number of cases in Asia [3]. Anemia prevalence in a country is inversely correlated with socio-economic development ranging from 9% in highly developed countries to 43% in the developing world [1,3]. Anemia is a significant global health burden as it negatively affects cognitive and motor development in children and results in fatigue and low productivity in the general population [1,4]. Children, women of a reproductive age, and the elderly are the most susceptible population segments [3].

The most common global risk factor for anemia is iron deficiency from malnutrition, malabsorption, or both, leading to a decreased production of red blood cells [2]. Other anemia risk factors include acute and chronic infections (inflammation), other micronutrient deficiencies (including folate, vitamin B12, and vitamin A), and genetic conditions such as thalassemia [5]. Circulating hemoglobin is the clinical biomarker of anemia [5].

The Chinese Government commonly addresses anemia and insufficient iron intake through food fortification. As soy sauce is used all over China and the risk of overconsumption is low due to its high salt content, the Chinese Government introduced iron-fortified soy sauce [6] and the fortification has subsequently proven effective in the Chinese population [7,8,9]. In 2002, the anemia prevalence in the Jiangsu province of eastern China was 18% for men and 32% for women [10]. However, it is difficult to identify individuals at high risk for anemia due to the complex relationships between anemia and the different nutritional intakes, in addition to other genetic and environmental factors including socio-economic and political factors [1]. Therefore, a single source of additional iron may not be a viable strategy in all segments of the population at risk, thus a more comprehensive analysis of the full nutritional and socioeconomic context is warranted.

The Jiangsu longitudinal cohort in Eastern China was set up to investigate the nutritional composition in the local population and its effects on public health. In this study, we sought to use a systematic approach to identify nutritional intake patterns of prevalent and incident anemia. First, we analysed univariate associations between the intake of a nutrient and anemia. In the second stage, we used a multi-variate data-mining framework based on the self-organizing map (SOM) to define representative subgroups of nutritional intake that were predictive of incident anemia. Population-based cohorts are unlikely to show an intrinsic clustering structure due to genetic diversity, environmental factors, and the wide age range, which makes conventional approaches less practical. We designed the SOM framework for these types of datasets to provide a more intuitive process of defining subgroups and their profiles [11,12]. Our results provide an important data-driven insight into the complex nutritional and demographic diversity that contributes to anemia susceptibility in different subpopulations.

## 2. Materials and Methods

### 2.1. Data Collection

This cohort study uses a subsample of the Chinese National Nutrition and Health Survey [13] in the Jiangsu Province in 2002 as the baseline. The follow-up data was collected in 2007; all reported incidence rates refer to a five-year follow-up period. Rural areas comprised three randomly selected towns in each of the following six counties: Jiangyin, Taicang, Suining, Jurong, Sihong, and Haimen, and urban areas included three randomly selected streets in the two large cities Nanjing and Xuzhou. In each rural town or city street, two villages or neighbourhoods were randomly selected and 90 households from each of them were selected at random. All family members in a household were invited to participate. A third of the households were queried on their dietary intake and all household members aged three years and above were invited to give fasting blood samples. Persons above 20 and up to 87 years of age were included. The study was approved by the Jiangsu Provincial Center for Disease Control and Prevention and all participants gave their written consent.

### 2.2. Primary Outcome and Data Availability

Anemia was defined as fasting blood hemoglobin below 13 g/dL for men and 12 g/dL for women [14,15]. Anemia status was available for 2849 participants at baseline (27.5% of the entire sample). A total of 737 women and 525 men had their anemia status available at both time points. These individuals still lived in the same household at the follow-up. The 1587 individuals lost to the follow-up were significantly younger (*p* < 0.001), had a higher baseline intake of iron (*p* = 0.001), and higher baseline levels of hemoglobin (*p* < 0.001) compared to the 1262 individuals that were followed up. Significantly more men than women were lost to follow-up (*p* < 0.001). There were no differences in the body mass index (BMI) (*p* = 0.30), baseline serum ferritin levels (*p* = 0.35), and total baseline calorie intake (*p* = 0.21) in both sets.

### 2.3. Study Design

**Figure 1:**
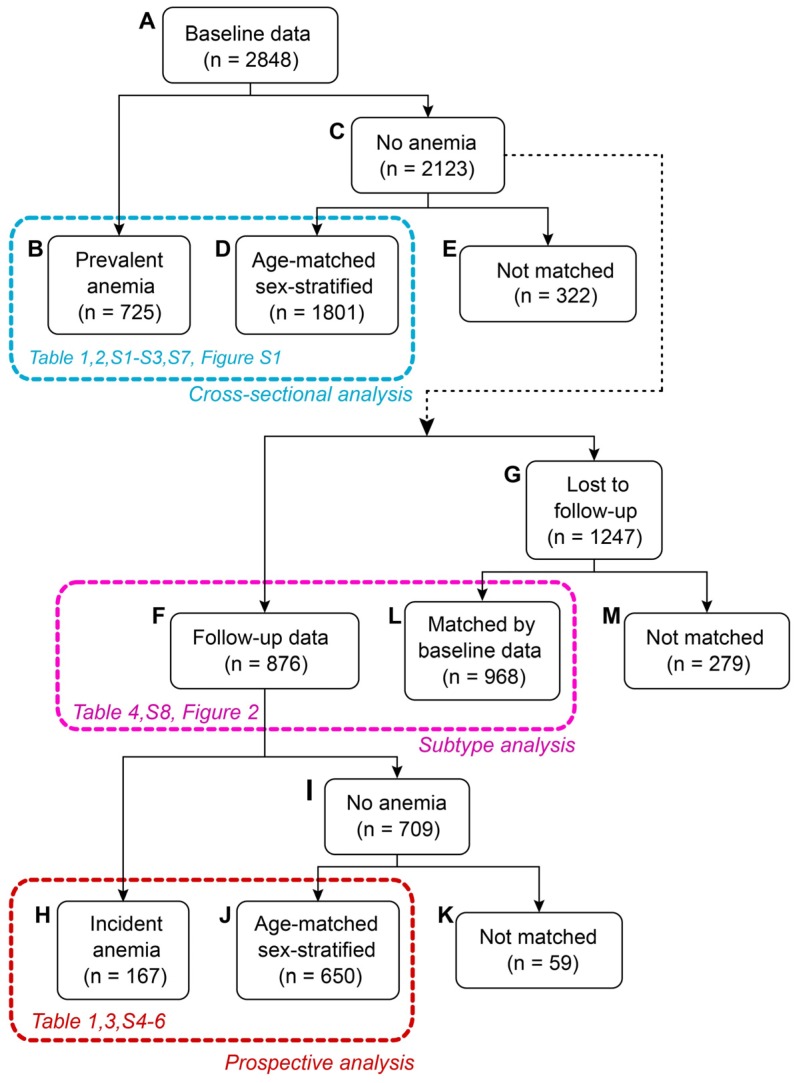
Selection of participants according to the study design. In total, nutritional data were available for 2848 participants (**A**). For the cross-sectional analyses, age-matched individuals with or without anemia were selected for sex-stratified comparison (**B**,**D**). Individuals without anemia at baseline (**C**), including those that could not be matched for the cross-sectional study (**E**), were eligible for the follow-up analyses. Follow-up data were available for 876 individuals (**F**) of whom 167 develop anemia (**H**) and 709 did not (**I**) during the follow-up period. For statistical comparisons, an age-matched and sex-stratified approach was applied (**H**,**J**), without the 59 individuals (**K**) who could not be matched with incident cases of anemia (**K**). Participants at risk who did not have follow-up data (**G**) were matched against the individuals with data (**F**) to create an independent baseline self-organizing map. The remaining individuals were not used for subtype analyses (**M**).

The exact sample numbers for statistical analyses are listed in Figure 1. There were 2848 individuals aged 20 years or above that had their baseline anemia status available and a usable baseline height measurement (Figure 1A). We defined subsets for baseline (Figure 1B,D) and prospective analyses (Figure 1H,J), respectively, and an extra subset for independent training of the subgroup models (Figure 1F,L). A matched case-control design was applied in univariate analyses (cross-sectional and prospective), whereas all individuals with sufficient data were used for evaluating the multivariable subgroups. To improve the phenotypic matching quality (Figure 1F,L), we applied a correlation network approach and grouped inter-correlated variables together (Table S7, Figure S1). We used principal component analysis to summarize the variation within each network module into a single score. These phenotypic scores were used for matching the subtype training set.

At baseline, there were 725 cases of anemia (Figure 1B). The 2123 individuals who did not have anemia were eligible to be included as controls (Figure 1C). We selected as many controls as possible from the eligible set without creating a significant difference (*p* > 0.05) in age distributions between the cases and controls. The matching was done separately for men and women. The final baseline subset comprised 725 individuals with anemia (Figure 1B) and 1801 matched controls (Figure 1D). For the prospective study, 876 individuals without anemia at baseline, and who were not lost to follow-up, were available (Figure 1F). There were 167 incident cases of anemia (Figure 1H). The remaining 709 individuals without anemia at follow-up were eligible to be included as controls (Figure 1I). The final prospective subset comprised 167 cases (Figure 1H) and 650 age-matched and sex-stratified controls (Figure 1J).

While 1247 individuals were lost to follow-up, their baseline data were usable as an independent training material for subtyping (Figure 1G). Furthermore, the subtyping framework did not require a case-control setting, therefore all individuals with follow-up data on anemia were available for evaluating the subgroups. To ensure that the subgroups based on the independent training set were compatible with the evaluation set, comprehensive matching based on the correlation structure of the baseline phenotypical information was performed (see Chapter 2.7. for further information). Furthermore, we ensured that the two sets showed no significant differences with regards to age, sex, baseline hemoglobin and ferritin, and baseline daily iron and calorie intake. The final training set comprised 968 matched individuals (Figure 1L), and the subgroup evaluation set included all 876 individuals with follow-up data who did not have anemia at baseline (Figure 1F).

### 2.4. Dietary Measurements

All food consumed by each individual participant over three consecutive days including one weekend day was recorded by health professionals [16]. The intake data in this study included information on staple foods (rice, wheat, other cereals, meat, vegetables, and fruit) and animal fat, vegetable oil, milk, eggs, soy sauce, salt, nuts, cakes, and alcohol intake. Total meat intake was defined as pork, poultry, fish, organ meat intake, and other meat intake that does not belong to any of the groups previously mentioned. Total vegetable consumption was defined as tubers, dark-leaved vegetables, light-leaved vegetables, salted vegetables, dark legumes, and other legumes intake. These food intake data were analysed using the Chinese Food Composition Table to derive macro- and micronutrient intakes [16]. The micronutrient data included the daily intakes of copper, iron, magnesium, manganese, phosphorus, potassium, selenium, vitamin A, B1, B2, C, PP (Niacin), retinol, and zinc. Vitamin E was split into α-, β-, and γ-tocopherol intake. Macronutrient variables included total daily calories, fat, carbohydrate, protein, and fibre intakes. Nutritional intake data were calibrated including micro- and macronutrient measures using the individual’s height. Height was chosen for calibration instead of total calories intake to preserve the shared variation between diet and obesity. The intakes of men and women were calibrated separately. According to the Chinese guidelines [17], low iron intake was defined as below 20 mg/day for women between the ages of 18 and 49 years, and below 12 mg/day for everyone else. To convert volume to weight, soy sauce density was defined as 1.12 g/mL [18].

### 2.5. Biochemistry and Clinical Data

Hemoglobin (cyanmethemoglobin method) and plasma glucose (hexokinase colorimetry) were measured at the local Centers for Disease Control and Prevention. Serum ferritin was measured in the National Center for Disease Control and Prevention in Beijing. Waist circumference was measured midway between the inferior margin of the last rib and the crest of the ilium in a horizontal plane, and height was measured without shoes to the nearest mm with a stadiometer. Hypertension was defined as the use of antihypertensive medication, systolic blood pressure above 140 mmHg, or diastolic blood pressure above 90 mmHg derived from the mean of two blood pressure measurements after 5 min of sitting by a mercury sphygmomanometer on the right upper arm. Type 2 diabetes was defined as having a fasting plasma glucose level above 7.0 mmol/L. In general, treatment for any diagnostic criterion automatically fulfilled it. Individuals with a BMI above 28 kg/m^2^ were considered obese. Metabolic syndrome was defined according to the 2005 International Diabetes Federation definition [19]: waist circumference ≥90 cm for men, ≥80 cm for women (central obesity); and at least two of the following conditions: triglycerides ≥150 mg/dL; fasting glucose ≥100 mg/dL (5.6 mmol/L); hypertension (systolic blood pressure (BP) ≥130 or diastolic BP ≥85 mmHg); or cholesterol in high density lipoproteins (HDL) <40 mg/dL in men, <50 mg/dL in women. 

### 2.6. Socio-Economic Factors

Education was defined as *low* (illiteracy, primary school), *medium* (junior middle school), and *high* (at least high middle school). Occupation was divided into *manual* and *non-manual* based on twelve occupational categories. Smoking was defined as *no smoking*, *light smoking* (1–19 cigarettes per day), and *heavy smoking* (20 and more cigarettes per day). Income was assessed by household income and number of people living in a household. It was defined as *low* (<1999 Yuan/person), *medium* (2000–4999 Yuan/person), and *high* (>5000 Yuan/person). We transformed these three income levels into three numbers: 1000 for the *low* level, 3500 for the *mid* level, and 5000 for the *high* level. Consequently, household income was treated as a numerical attribute in our analysis for easier interpretation.

### 2.7. Statistical Analysis

The univariate analyses at baseline and follow-up were conducted separately for age-matched men and age-matched women. For the main text, the sex-specific results were combined by calculating the number-weighted means of the descriptive statistics. Prior to statistical comparisons, we used linear regression to remove any co-variation with age from quantitative data. Confounding age-effects on binary variables were controlled by adding age as a co-variate in logistic models of anemia. False discovery rates were calculated using the *stats* package from *R*. *p*-values were combined using Fisher’s method as implemented in the *R* package *metap* [20]. We performed a logistic regression analysis with all traits that were significantly associated with incident anemia (Table S6). The model was adjusted for sex, age, body mass index, smoking, education, income, manual work, total calories, fat, fibre, and alcohol intake.

Spearman’s pair-wise correlations between all measures in the baseline cohort were estimated separately for women and men and then combined, weighted by the fraction of female and male participants (Table S7). The results yielded a network (Figure S1) where the nodes represent variables and the weighted links represent the sex-adjusted correlation coefficients. The network was pruned in order to focus only on the strongest connections by a previously published spanning tree algorithm [21]. An in-house implementation of the agglomerative community detection algorithm was used to define modules of correlated traits [22]. To summarise the aggregate information captured by the network modules, the first principal component [23] was extracted as a single representative score for each module.

Multivariable subtypes were defined with the self-organizing map (SOM) framework [11,12,24,25,26]. Briefly, the SOM framework creates a two-dimensional representation of a multi-dimensional dataset, that is, a “map” of the individuals. On the map, individuals who are far apart are different with respect to nutritional intake, whereas individuals at the same map location share a similar overall profile. Next, the software estimates statistics and paints the subdivisions on the map according to how much anemia (or other characteristic) is observed in the resident subpopulation. In the final stage, we use the map colorings to determine areas (i.e., subgroups) with high or low anemia risk. A more detailed introduction to the SOM concept is available as an online supplement within a previous paper [24].

**Figure 2:**
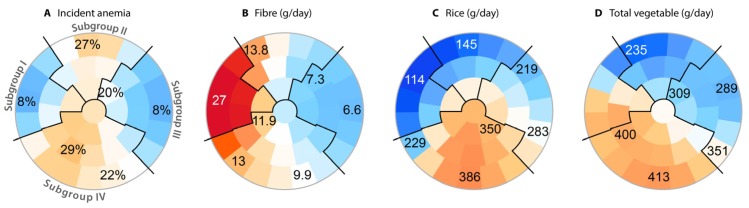
The data-driven self-organizing map (SOM) with subgroup boundaries, coloured according to subtype averages of four different traits. On each colouring from **A**–**D**, a particular individual resides in the same place, as one would expect for colourings of a geographical map of a city, for example. The colours were created by averaging over the local residents within the subdivisions that each represent a single data-driven subtype. The numbers on the map indicate the numerical averages for selected subtypes.

The SOM was evaluated for all individuals that were followed up and did not have anemia at baseline (Figure 1F) and was trained with a subset of individuals that were lost to follow-up and did not have anemia at baseline (Figure 1L). This training subset closely matched the ones that were followed-up. Matching was based on the first two principal components for each network module (Figure S1) as these components summarise the aggregate information from the network modules [27]. We selected the closest matching pairs that showed no significant difference (*p* > 0.05) in age, sex, baseline hemoglobin and ferritin levels, and baseline daily calories and iron intake. To eliminate confounding effects from gender, the SOM training data were first split by sex, all continuous variables were then converted into ranks within the genders, respectively, and finally, the two groups were pooled back together before applying the SOM algorithm. The pattern for incident anemia at the five-year follow-up suggested four subgroups (Figure 2). The mean values for each subgroup, together with 95% confidence intervals, were estimated by bootstrapping with 10,000 simulations.

## 3. Results

The baseline characteristics of the study populations (Figure 1B,D,H,J) are listed in Table 1. Overall, the cross-sectional baseline set and the prospective data were similar with respect to sex (61% women vs. 58% men), age (49 years vs. 50 years), and BMI (24 kg/m^2^ in both cohorts). The prevalence of anemia was 29% at baseline. The follow-up set only included individuals that did not have anemia at baseline. Consequently, individuals at follow-up had higher baseline levels of hemoglobin (+7 g/dL, *p* < 0.001). These individuals also had higher baseline concentrations of serum ferritin (+7 ng/mL, *p* = 0.03). In addition, fewer people in the follow-up cohort had a high education (‒4%, *p* = 0.03) and fewer lived in urban areas (‒9%, *p* < 0.001).

### 3.1. Univariate Associations with Prevalent and Incident Anemia

Table 2 lists univariate differences between individuals with and without anemia at baseline. Individuals with anemia at baseline had a lower total calorie intake (−103 kcal, *p* < 0.001) and they ate more rice (+38 g/day, *p* < 0.001), but less wheat (−52 g/day, *p* < 0.001). The total meat intake was increased compared to the controls (+21 g/day, *p* < 0.001). Prevalent anemia was associated with a 2 mg/day lower iron intake (*p* < 0.001) and a slightly lower fibre intake (−2 g/day, *p* < 0.001). In general, individuals with anemia were more likely to be female (67%, *p* < 0.001), live in southern Jiangsu (61%, *p* = 0.003), and have a higher household earning (+348 Yuan/person, *p* < 0.001). Living in southern Jiangsu remained significantly associated with prevalent anemia in a logistic regression model that was adjusted for sex and age. Smoking was not associated with prevalent anemia. Additional details, including the results for each sex, are available in Supplementary Tables S1–S3.

Incident anemia (Table 3) was associated with a higher rice intake (+50 g/day, *p* < 0.001). Although there was no difference in total baseline meat and vegetable intake, individuals with incident anemia consumed less organ (−4 g/day, *p* < 0.001) and other meat (−6 g/day, *p* < 0.001) and less legumes (−7 g/day, *p* < 0.001) at baseline. Geographical residence, smoking, income, and total calories and fibre intake were not predictive of anemia (*p* > 0.05). Individuals with incident anemia were more likely to have a low education level at baseline (65% vs. 50%, *p* = 0.004). This association with incident anemia remained significant in a logistic regression analysis that was adjusted for sex and age. Additional details including the results for each sex are available in Supplement Tables S4–S6. In a logistic regression analysis with all traits that have been univariately associated with incident anemia from Table S6, hemoglobin (*p* < 0.001) and rice intake (*p* = 0.01) (together with the intakes of salted vegetables and β-tocopherol and exercise) showed a significant association.

Men with incident anemia had lower baseline concentrations of triglycerides (‒35 mg/dL difference) and hemoglobin (‒7 g/dL) compared to men who did not develop incident anemia (*p* < 0.001). No associations were found in women (*p* > 0.5). Please see Supplementary Tables S4 and S5 for details. In individuals older than the median of 48 years, incident anemia was associated with lower triglycerides (‒25 mg/dL, *p* < 0.001) and less sedentary time (‒24 min/day, *p* = 0.005) at baseline. In younger individuals, incident anemia was associated with consuming less organ meat (‒6 g/day) and fruit (‒32 g/day) at baseline (*p* < 0.001).

### 3.2. Nutritional Subgroups with High or Low Risk of Anemia

To identify vulnerable subgroups, we applied a multivariable subtyping algorithm. Specifically, we used a visual inspection of the SOM subtypes to summarize the Jiangsu dataset by a limited number of subgroups. The subtypes are automatically organized on a two-dimensional map in a way that adjacent subtypes show only minor differences, whereas subtypes on the opposite edges of the map are substantially different. This allowed us to stratify the spectrum of nutritional profiles in the original dataset into only four representative subgroups (Figure 2, Table 4 and Table S8).

Subgroup I, located in the left quadrant of the SOM (Figure 2), was associated with a mean anemia incidence of 10% (95% confidence interval (CI95): 6–16%) compared to 19% in the overall evaluation set in the five-year follow-up period (Figure 2A, please note that the numbers on the map indicate the averages at specific locations on the map, not across the entire quadrants). In the upper quadrant, Subgroup II was associated with a 25% (CI95: 19–32%) incidence during the same period (Figure 2A). We then examined the baseline data behind these two subgroups to develop a simplified description: Subgroup I was characterised by a high fibre intake (mean 27 g/day, CI95: 24–29 g/day) (Figure 2B) compared to a mean intake of 12 g/day in the entire evaluation set. On the other hand, Subgroup II was characterized by a low vegetable intake (mean 286 g/day, CI95: 265–309 g/day) (Figure 2D) compared to the evaluation set (366 g/day). For these reasons, we designated the subgroups as High Fibre Intake (I) and Low Vegetable Intake (II).

Subgroup III was located on the right side of the SOM, was associated with an anemia incidence of 10% (CI95: 7–14%, Figure 2A), and was characterized by a low fibre intake (7 g/day, CI95: 7–8 g/day) (Figure 2B). Subgroup IV was located in the bottom part of the map, with the highest incidence rate of 27% (CI95: 22–31%, Figure 2A). It was characterized by a high rice intake (372 g/day, CI95: 360–385 g/day) (Figure 2C) compared to the overall evaluation set mean of 272 g/day. Hence we designated these two subgroups as Low Fibre Intake (III) and High Rice Intake (IV).

Compared to the High Fibre subgroup (I), the Low Vegetable subgroup (II) consumed considerably more meat (+33 g/day) and rice (+56 g/day) and less wheat (−120 g/day), vegetables (−148 g/day), and iron (−14 mg/day). No individual in Subgroup I consumed iron below the recommended threshold, but 15% (CI95: 10–25%) of individuals in Subgroup II consumed less than the recommended amount. Both subgroups lived mainly in Northern Jiangsu (97% and 66%). The High Rice subgroup (IV) consumed considerably more rice (+101 g/day) and iron (+8 mg/day) compared to the Low Fibre subgroup (III). Both subgroups had a similar intake of meat and wheat (overlapping CI95). However, Subgroup IV had a lower income compared to Subgroup III (−170 Yuan/person). Both subgroups lived mainly in Southern Jiangsu (98% and 83%).

## 4. Discussion and Conclusions

Anemia affects a significant proportion of the global population, negatively affecting cognitive and motor development in children and resulting in fatigue and low productivity in the general population [1,4]. Nutritional intake (of iron) is a major modifiable factor in mitigating the public health impact of anemia. In this study, we analysed a large prospective cohort from the Jiangsu province in Eastern China with detailed baseline data on nutrient intake. Univariate analyses were performed on cross-sectional and prospective subsets of the available data, and we replicated findings from previous studies. Multivariable analyses enabled us to summarize the spectrum of nutritional and socio-economic profiles by four subgroups: two of the subgroups (High Fibre and Low Vegetable) were primarily from the less affluent North, and showed the expected inverse association between iron intake and anemia. However, the predominantly Southern subgroups were surprising: the Low Fibre subgroup showed a lower anemia incidence, yet also a lower iron intake compared to the High Rice subgroup. This finding is particularly relevant with respect to iron fortification as a potential preventative measure.

Prevalent anemia was associated with a lower iron intake (Table 2). It was also associated with the intakes of grains and meat and household income. Low iron intake is a risk factor for anemia [3] and its association was further supported by grain intake, as different grains yield different amounts of iron. As expected, rice (lower iron yield) was positively and wheat (higher iron yield) inversely associated with anemia [28]. Interestingly, meat, which is a good source of iron [29], was positively associated with anemia. Individuals with anemia at baseline might have changed their dietary habits to improve their iron intake. Low socio-economic background has been previously identified as another risk factor for anemia [1], but the opposite was true in the Jiangsu cohort (low income was associated with lower anemia prevalence in Table 2). The result was most likely confounded by the difference in nutritional patterns and income levels between the northern and southern parts of the province, as anemia is more prevalent in the economically more developed south [30,31] that also traditionally follows a rice-based diet as opposed to a wheat-based one in the north [32].

The five-year incidence of anemia was 19% in the Jiangsu cohort (38 new cases per 1000 person years). In another Chinese cohort from Deqing county, a higher incidence of 220 cases per 1000 person years was reported during a two-year follow-up of 847 individuals [33]. Both cohorts were of a comparable average age and sex-distribution, but the population base was different. The Deqing was limited to one county whereas Jiangsu covered a whole province, which may explain the discrepancy. Lower incidence rates were observed in a Korean cohort (24 cases per 1000 person years) [34] and in an Italian cohort (22.5 cases per 1000 person years), both of older individuals [35]. In a population-based German cohort, the incidence was 5.8 cases per 1000 person-years [36]. Due to observational bias in the elderly and differences between countries and ethnicities, it is difficult to assess if the higher Jiangsu rates are compatible with a specific cohort from elsewhere but, overall, the incidence we observed is realistic within the range of reported results, and therefore likely to be accurate given the design of the Jiangsu cohort. Importantly, our observations also demonstrate that anemia remains a significant public health challenge in Jiangsu.

Previous studies on the Jiangsu cohort have reported prevalent anemia [10,32,37,38,39], and limited parts of the prospective dataset within the incidence of multimorbidity or persistent anemia at follow-up [16,40]. This study is the first to systematically investigate incident anemia in Jiangsu. Rice and wheat intake were associated with the incidence of multimorbidity (including anemia) in a previous study of the Jiangsu cohorts [40]. However, wheat or iron intake did not predict anemia status as a specific morbidity in this study despite cross-sectional associations, which is supported by a similar previous analysis [16]. A possible explanation may be that the average baseline iron intake was above the recommended Chinese intake even for people who developed anemia during the follow-up (24 mg/day intake vs. 20 mg/day for pre-menopausal women and 12 mg/day for the general population). Therefore, other factors beyond iron intake may be involved in the development of anemia in these individuals.

Multivariable profiling with the SOM led to four subgroups that were named according to characteristic nutritional features. There was one subgroup with a characteristically high fibre intake (I) and one with a low one (III). Fibre is a known inhibitor of iron absorption [29], but both subgroups had a low incident anemia rate. A possible explanation is that the subgroup High Fibre also had the highest intake of iron of all subgroups. In addition, there was also a prominent north-south divide: the two predominantly northern subgroups (High Fibre (I) and Low Vegetable (II)) behaved as expected (low socio-economic status and low iron intake predicted anemia), whereas the predominantly southern subgroups (Low Fibre (III) and High Rice (IV)) were more complicated. From a translational perspective, these findings indicate that established strategies to reduce the public health impact of anemia in northern Jiangsu are likely to work well, whereas more research is warranted in the south. Jiangsu should be treated as two entities economically because of inherent disparity [30] and our study suggests that this applies to incident anemia as well.

Iron-fortified soy sauce is used in China to prevent anemia and it can be considered as a one-fits-all approach. In this study, baseline soy sauce intake did not include additional iron as the iron-fortification of soy sauce was approved in 2002, and therefore not yet fully implemented at the time of data collection [41]. However, the potential role of the fortification can be assessed by estimating the expected increase in iron intake. If we compare no fortification against the high NaFeEDTA (ferric sodium ethylenediaminetetraacetate) fortification scheme (+4 mg/mL added iron) from the literature [6], the maximum theoretical decrease in the prevalence of low iron intake would be −9% in the evaluation set, with the greatest decreases of −16% and −13% in the Low Fibre (III) and Low Vegetable (II) subgroups, but a limited or no benefit for High Rice (IV) or High Fibre (I) subgroups (−6% and 0% decrease). Therefore, iron fortification could potentially help address the high incidence of anemia in the Low Vegetable subgroup, but it might not be effective to lower the high incidence within the High Rice subgroup.

According to the theoretical predictions, the average added supplemented iron intake in the evaluation subset would be approximately +38 mg/day. A supplemented intake of more than +30 mg/day increased the odds for high iron stores more than four-fold [42]. The health effects of too much iron remain controversial, but some evidence suggests that high body iron stores increase the risk of chronic diseases such as heart disease, cancer, and diabetes [42]. Internationally reported NaFeEDTA fortification levels of soy sauce are typically lower (0.25 mg/g) (Table 7.10 in [43]). However, a study based on the same cohort has found an association of low and high iron intake (represented as a percentage of the recommended intake) with an increased risk for all-cause mortality in women [44]. To this backdrop, better identification of individuals who are at risk of anemia primarily because of iron intake (e.g., via subgroup analyses) becomes even more important.

The collection of detailed household food intake is a methodological strength of this study. Furthermore, the Jiangsu cohort reflects the geographic and economic diversity within the province [15] and the results on prevalent anemia are likely to reflect the respective age segments in the local population. The data on incident anemia were collected from adults who did not have anemia at baseline, so the conclusions may not be applicable to individuals who developed anemia earlier in their life. To maximize the accuracy of the statistical analyses, matched cases and controls were used for all univariate tests, and a sophisticated design with an independent training set was used for the multivariable analysis. In particular, we were able to use most of the individuals from the baseline despite the lack of follow-up data. One limitation of the study is that we only used nutritional data from baseline. The Chinese Health and Nutrition Survey [13] reported that diet patterns were stable between 2002 and 2007 [45], therefore we expect our findings to be relevant for a large part of the Eastern Chinese population. Another limitation of the study is that potential confounders of anemia were not included in the study, for example, infectious disease, environmental pollution, medication history, or time spent away from home during follow-up. We did not have detailed data on the bioavailability of iron, micronutrient deficiencies (e.g., folate and vitamin B12), or genetics, and therefore, caution is warranted against a causal interpretation of our findings. Thalassemia was not a major cause of anemia in the study area (prevalence < 0.1%) [10].

Complete separation between the training and evaluation data is a strength of the SOM framework, since the design prevents overfitting. For instance, we have compared an SOM constructed in 2008 against data that became available in 2015 with excellent reproducibility [11]. On the other hand, the SOM was not designed for diagnostic modelling where an accurate gold-standard is available for training, and we recommend using supervised methods and replication cohorts in those studies. Transparency is another strength of the SOM approach: the process of defining subgroups and investigating their profiles is user-driven and open for constructive criticism from other human observers (i.e., the map colorings), whereas other clustering algorithms are black boxes that output the classification results without the explicit involvement of the researcher, or without descriptive statistics on the amplitude of the data patterns.

This study highlights the inherent natural heterogeneity of nutritional profiles and how they are associated with incident anemia in a population-based setting. Our subgroup study shows how nutritional and economic differences between the northern and southern part of Jiangsu predict differences in incident anemia. While the predominantly northern subgroups fit with the global consensus of anemia risk factors (low socio-economic status and low iron intake), incident anemia in the two mostly southern subgroups shows different associations. In particular, there are theoretical indications that iron fortified soy sauce (the prevalent public health intervention) may not address anemia risk equally well in all subgroups, and may even have unintended adverse consequences. Therefore, different strategies may be needed to limit the health impact from anemia across the population segments in China.

## Figures and Tables

**Table 1:** Baseline characteristics of the datasets in the cross-sectional analyses (Figure 1B,D) and the prospective analyses (Figure 1H,J). ^1^

	Cross—Sectional Subset	Prospective Subset	*p*-Value
Participants	2526	817	N/A
Female	61%	58%	0.16
Anemia prevalence	29%	0%	<0.001 *
Age (years)	49 ± 14	50 ± 13	0.05
BMI (kg/m^2^)	24 ± 4	24 ± 3	0.04
Total cholesterol (mg/dL)	149 ± 37	154 ± 45	<0.001 *
High-density lipoprotein cholesterol (mg/dL)	51 ± 12	51 ± 11	0.71
Triglycerides (mg/dL)	99 ± 73	109 ± 68	<0.001 *
Hemoglobin (g/dL)	132 ± 18	139 ± 12	<0.001 *
Serum ferritin (ng/mL)	94 ± 84	101 ± 85	0.03 *
Low education level	52%	53%	0.44
High education level	15%	11%	0.01 *
Urban residency	24%	15%	<0.001 *

^1^ Means and standard deviations are reported when available. * False discovery rate of less than 5%. BMI, body mass index.

**Table 2:** Baseline associations (sex-stratified, age matched, and adjusted) with prevalent anemia (Figure 1B,D). ^1^

	No Anemia	Anemia	*p*-Value
Number (%)	1801 (71%)	725 (29%)	
Age (years)	48 ± 14	50 ± 15	0.04
Female	59%	67%	<0.001 *
BMI (kg/m^2^)	23.8 ± 3.5	22.9 ± 3.5	<0.001 *
Hemoglobin(g/dL)	140 ± 13	113 ± 11	<0.001 *
Ferritin (ng/mL)	96 ± 82	86 ± 77	0.007 *
Rice (g/day)	242 ± 150	280 ± 130	<0.001 *
Wheat (g/day)	127 ± 152	75 ± 116	<0.001 *
Total meat (g/day)	135 ± 109	156 ± 100	<0.001 *
Organ meat (g/day)	5 ± 17	6 ± 22	0.27
Other meat (g/day)	7 ± 22	6 ± 18	0.27
Total vegetable (g/day)	346 ± 179	333 ± 161	0.19
Legumes (g/day)	15 ± 23	12 ± 19	0.02 *
Fibre (g/day)	13 ± 11	11 ± 9	<0.001 *
Soy sauce (g/day)	11 ± 16	10 ± 14	0.44
Total calories (kcal/day)	2326 ± 671	2223 ± 619	<0.001 *
Iron (mg/day)	26 ± 11	24 ± 11	<0.001 *
Southern Jiangsu ^†^	54%	61%	0.003 *
Low education level ^†^	51%	54%	0.17
Light smoking ^†^	12%	13%	0.93
Heavy smoking ^†^	13%	11%	0.34
Income (Yuan/person)	3176 ± 1674	3524 ± 1497	<0.001 *

^1^ Means and standard deviations are reported when available. Statistical significance was adjusted for sex. * False discovery rate below 5%. ^†^ not adjusted for age.

**Table 3:** Baseline associations (sex-stratified, age matched, and adjusted) with incident anemia during a five-year follow-up (Figure 1H,J). ^1^

	No Anemia	Incident Anemia	*p*-Value
Number (%)	650 (80%)	167 (19%)	
Age (years)	49 ± 13	52 ± 11	0.06
Female	56%	65%	0.07
BMI (kg/m^2^)	23.8 ± 3.3	23.8 ± 3.3	0.56
Hemoglobin (g/dL)	140 ± 13	137 ± 10	<0.001 *
Ferritin (ng/mL)	101 ± 81	100 ± 92	0.77
Rice (g/day)	262 ± 139	312 ± 152	<0.001 *
Wheat (g/day)	107 ± 145	88 ± 123	0.14
Total meat (g/day)	149 ± 111	143 ± 111	0.76
Organ meat (g/day)	6 ± 18	2 ± 12	<0.001 *
Other meat (g/day)	8 ± 24	2 ± 13	<0.001 *
Total vegetable (g/day)	365 ± 181	367 ± 181	0.07
Legumes (g/day)	16 ± 24	9 ± 16	<0.001 *
Fibre (g/day)	12 ± 10	12 ± 8	0.53
Soy sauce (g/day)	11 ± 16	10 ± 18	0.02
Total calories (kcal/day)	2338 ± 656	2437 ± 630	0.15
Iron (mg/day)	26 ± 9	24 ± 10	0.21
Southern Jiangsu ^†^	65%	67%	0.79
Low education level ^†^	50%	65%	0.004 *
Light smoking ^†^	13%	11%	0.87
Heavy smoking ^†^	16%	14%	0.95
Income (Yuan/person)	3395 ± 1672	3382 ± 1401	0.30

^1^ Means and standard deviations are reported when available. Statistical significance was adjusted for sex. * False discovery rate below 5%. ^†^ not adjusted for age.

**Table 4:** Subgroup-specific risk factors for incident anemia during five years of follow-up (Figure 1F,L). ^1^

	Full Eval. Set	High Fibre (I)	Low Veg. (II)	Low Fibre (III)	High Rice (IV)
Number (*n*)	876	143	160	250	323
Southern Jiangsu (%)	66	3 (1, 7) *	34 (27, 42) *	98 (96, 100) *	83 (79, 87) *
Incident anemia (%)	19	10 (6, 16) *	25 (19, 32)	10 (7, 14) *	27 (22, 31) *
Female (%)	54	59 (52, 67)	48 (41, 56)	59 (52, 65)	51 (46, 57)
Age (years)	49	46 (44, 48) *	52 (50, 54) *	50 (48, 52)	47 (46, 48) *
BMI (kg/m^2^)	24	23 (23, 24)	24 (24, 25)	24 (23, 24)	24 (23, 24)
Hemoglobin (g/dL)	140	143 (141, 145)	141 (139, 143)	139 (137, 141)	139 (138, 141)
Ferritin (ng/mL)	102	73 (63, 84) *	106 (92, 119)	110 (100, 121)	107 (98, 117)
Total calories (kcal)	2378	2760 (2656, 2869) *	1988 (1918, 2061) *	1954 (1901, 2007) *	2733 (2670, 2797) *
Rice (g/day)	272	137 (117, 158) *	193 (174, 212) *	271 (260, 282)	372 (360, 385) *
Wheat (g/day)	105	277 (246, 308) *	157 (137, 178) *	46 (38, 53) *	49 (40, 59) *
Total meat (g/day)	151	61 (49, 74) *	94 (81, 107) *	177 (165, 188) *	198 (185, 211) *
Total vegetable (g/day)	366	434 (392, 479) *	286 (265, 309) *	307 (294, 320) *	422 (404, 440) *
Legumes (g/day)	15	24 (20, 30) *	13 (10, 16)	13 (11, 15)	14 (11, 16)
Fibre (g/day)	12	27 (24, 29) *	11 (10, 11) *	7 (7, 8) *	11 (10, 12)
Soy sauce (g/day)	11	17 (13, 22) *	8 (7, 10) *	8 (7, 9) *	11 (10, 13)
Iron (mg/day)	26	35 (33, 36) *	21 (20, 22) *	20 (20, 21) *	28 (27, 29) *
Low iron (%)	12	0 (0, 0) *	15 (10, 21)	22 (17, 27) *	8 (6, 11) *
Urban residency (%)	15	4 (1, 8) *	24 (18, 31) *	26 (21, 32) *	6 (3, 9) *
Low education (%)	51	65 (57, 73) *	62 (54, 69) *	42 (36, 48) *	46 (40, 51)
Income (Yuan/person)	3405	1542 (1364, 1731) *	2741 (2484, 2994) *	4154 (3992, 4310) *	3984 (3847, 4115) *

^1^ Means and 95% confidence intervals are reported. * The mean of the entire evaluation set is outside the 95% confidence interval of the subgroup mean.

## Supplementary Materials

The following are available online at www.mdpi.com/2072-6643/9/10/1153/s1, Table S1: Comparison of baseline characteristics between women with and without anemia at baseline; Table S2: Comparison of baseline characteristics between men with and without anemia at baseline; Table S3: Sex-stratified comparison of baseline characteristics (mean ± standard deviation for numerical traits, percentages for binary traits) by merging the separate results for age-matched women and age-matched man with and without anemia at baseline; Table S4: Comparison of baseline characteristics between women with and without incident anemia at follow-up; Table S5: Comparison of baseline characteristics between men with and without incident anemia at follow-up; Table S6: Sex-stratified comparison of baseline characteristics (mean ± standard deviation for numerical traits, percentages for binary traits) by merging the separate results for age-matched women and age-matched man with and without incident anemia at follow-up; Table S7: Sex-stratified correlation coefficients of baseline measures; Table S8: Comparison between the four subgroups for five-year incident anemia at follow-up and the evaluation set; Figure S1: Correlation structure of the baseline subset. The nodes represent variables and the edge width indicates the strength of the correlation (thicker means stronger). Only topologically important edges are depicted: the strongest pair-wise correlations within a module are included, whereas the edges between any two modules are collapsed into a single grey line in the background.
