# Supplementary figures and images for: Multivariable Analysis of Nutritional and Socio-Economic Profiles Shows Differences in Incident Anemia for Northern and Southern Jiangsu in China
Source: Nutrients. 2017 Oct 21;9(10):1153. doi: 10.3390/nu9101153 (PMC5691769; doi:10.3390/nu9101153)

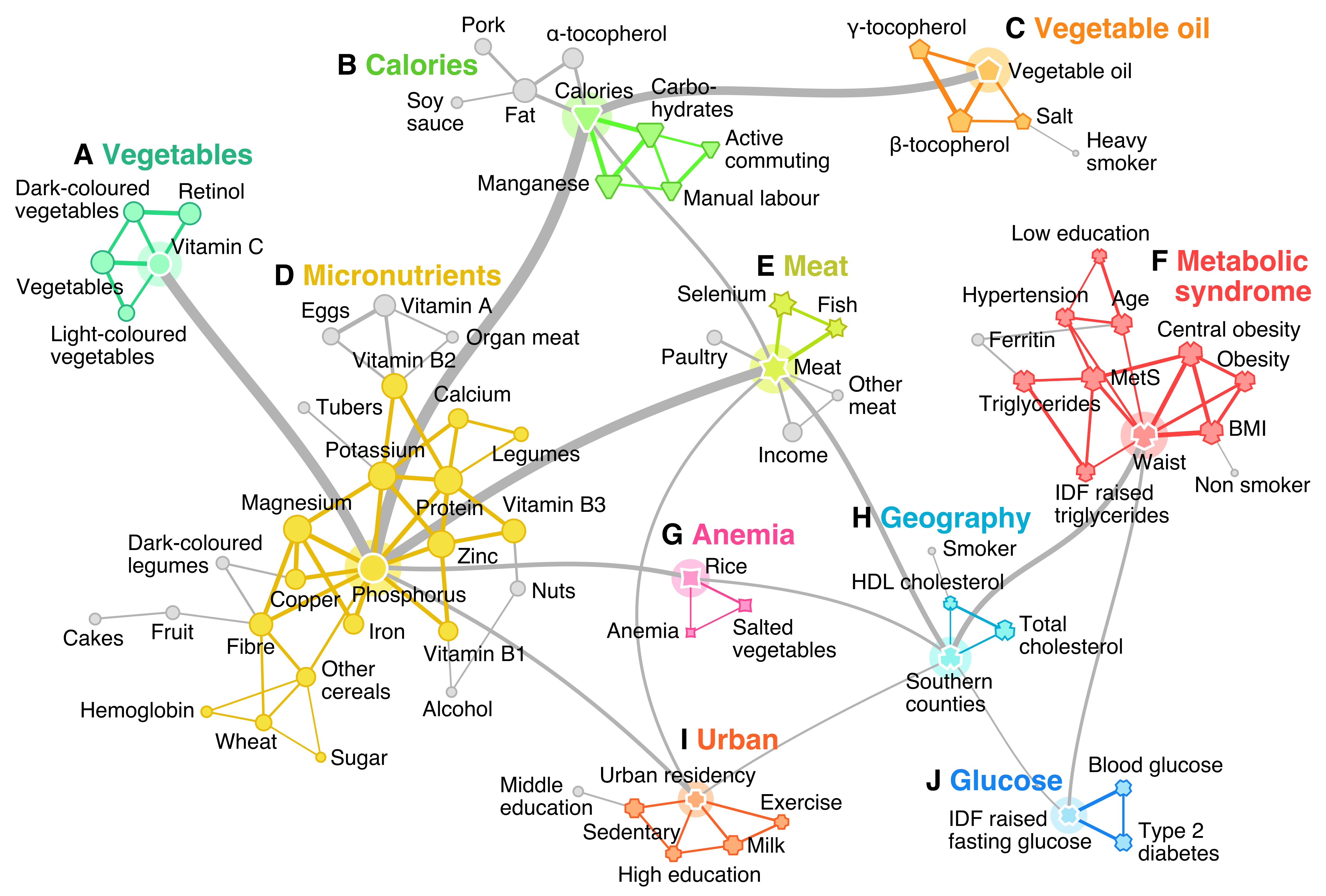

Supplement: Supplementary file 1 [file nutrients-09-01153-s001.zip › FigureS1.png]
